# Motor and Linguistic Linking of Space and Time in the Cerebellum

**DOI:** 10.1371/journal.pone.0007933

**Published:** 2009-11-20

**Authors:** Massimiliano Oliveri, Sonia Bonnì, Patrizia Turriziani, Giacomo Koch, Emanuele Lo Gerfo, Sara Torriero, Carmelo Mario Vicario, Laura Petrosini, Carlo Caltagirone

**Affiliations:** 1 Dipartimento di Psicologia, Università degli Studi di Palermo, Palermo, Italy; 2 Fondazione Santa Lucia IRCCS, Roma, Italy; 3 Clinica Neurologica, Università di Roma Tor Vergata, Rome, Italy; 4 Dipartimento di Psicologia, Università degli Studi di Roma La Sapienza, Rome, Italy; Harvard Medical School, United States of America

## Abstract

**Background:**

Recent literature documented the presence of spatial-temporal interactions in the human brain. The aim of the present study was to verify whether representation of past and future is also mapped onto spatial representations and whether the cerebellum may be a neural substrate for linking space and time in the linguistic domain. We asked whether processing of the tense of a verb is influenced by the space where response takes place and by the semantics of the verb.

**Principal Findings:**

Responses to past tense were facilitated in the left space while responses to future tense were facilitated in the right space. Repetitive transcranial magnetic stimulation (rTMS) of the right cerebellum selectively slowed down responses to future tense of action verbs; rTMS of both cerebellar hemispheres decreased accuracy of responses to past tense in the left space and to future tense in the right space for non-verbs, and to future tense in the right space for state verbs.

**Conclusions:**

The results suggest that representation of past and future is mapped onto spatial formats and that motor action could represent the link between spatial and temporal dimensions. Right cerebellar, left motor brain networks could be part of the prospective brain, whose primary function is to use past experiences to anticipate future events. Both cerebellar hemispheres could play a role in establishing the grammatical rules for verb conjugation.

## Introduction

Time perception can be distorted by a number of factors [1]. Spatial attention is one of such factors, able to affect the perception of time as well as of other magnitudes [2]. Indeed, recent studies suggested that representation of elapsing time is likely to be visuospatial in nature. Vicario et al. [3] used optokinetic stimulation as a technique to induce manipulation of spatial attention before time judgement tasks of sub-second intervals. They found that moving attention to the right led to overestimation of time intervals, while moving attention to the left induced underestimation of time intervals.

These directional biases in time perception argue for a mental linear representation of time intervals, with low intervals associated with left side space and higher intervals with right-side space [4]. The same authors [5] showed that lateralised presentation of visual stimuli biased their perceived duration according to the side of space where they were encoded. Right-sided visual stimuli were overestimated as compared with left-sided visual stimuli. The same pattern of results was recently reported in healthy subjects in whom directional biases of spatial attention were induced by prismatic adaptation procedure [6]. Specifically, rightward prismatic deviation, inducing leftward attentional biases, induced time underestimation, while leftward prismatic deviation, inducing rightward attentional biases, induced time overestimation. In the same strand of research, Vallesi et al. [7] documented spatial-response compatibility in temporal tasks requiring to respond to short vs. long intervals. Subjects were faster in responding to short intervals in the left space, whereas they were faster in responding to long intervals in the right space. According to the authors, this left-to-right directionality seems to be a consistent feature of how the cognitive system represents ordered material such as time, space, numbers. Therefore, the relation linking time and space perception could be similar to that between numbers and space processing, exemplified by the metaphor of mental number line [8].

In the present study we asked whether the mental time line metaphor could be extended to wide abstract concepts such as past and future.

Santiago et al. [9] reported that judgments of the past or future reference of written words are affected by spatial characteristics. Responses are faster when past and future time are mapped onto left and right keys, respectively, than when the opposite mapping is used. Moreover, words are processed faster and more accurately when they are presented on the screen side that is congruent with their temporal meaning: past words on the left, and future words on the right space. These findings would suggest that even the time of linguistic stimuli could be processed in both spatial and propositional formats.

An assumption underlying the present study is that this process of mapping the time of a verb onto spatial formats is influenced by motor imagery activated by simple reading of a verb describing an action. Behavioural and neurophysiologic studies documented the effects of linguistic processing on the activity of motor areas: reading of sentences associated with motor experiences activates areas involved in action execution [10]; processing of action verbs recruits motor areas in the brain and influences motor cortical excitability [11]–[12]. Such facilitation of motor cortical areas is influenced by temporal aspects of the action [13]–[16]. Motor cortical facilitation is maximal during observation of the initial and middle phases of a movement [16]. Activation of premotor cortices is also influenced by observation of logic sequences of non-biological events [17]–[20]. Dorsal areas of premotor cortex are activated when temporally predictable stimuli require a motor response, suggesting that the primary purpose of temporal expectation is to optimise prospective motor behaviour [21]. These studies suggest that motor activation could be related to the anticipation of future states. On the other hand, any differential recruitment of motor areas in the brain depending on the time of linguistic stimuli has never been investigated.

Several lines of evidence indicate the cerebellum as a good neural substrate for this process: cerebellar hemispheres are critically involved in the comprehension of actions as well as in observational learning; cerebellar hemispheres are involved in processing of time intervals, especially in the sub-second range, which is critical for comprehension of others actions [22]; cerebellar hemispheres are involved in processing of linguistic stimuli; the right cerebellar hemisphere, in addition to the left premotor cortex, is activated during mental representation of future states [23].

In a first series of experiments, we explored the contribute of spatial factors in time processing (past vs. future) using verbs and modifying their tense to monitor the specificity of semantic motor activations for spatial representation of past and future. In another experiment we analyzed the role of the cerebellum in this linguistic task using a repetitive transcranial magnetic stimulation (rTMS) interference approach in a group of healthy subjects.

## Materials and Methods

### Experiment 1

#### Subjects

Twenty-four healthy participants (age-range 20–30 years; education level: 12 years) took part in this study. All were right-handed, native Italian speakers. All subjects gave written informed consent for participation in the study, that was approved by the ethic committee of the Santa Lucia Foundation (Prot. CE-AG4-PROG.187-57).

#### Stimuli

Stimuli were divided in 3 lists. Each list included: 10 action verbs, 10 state verbs and 10 non-verbs. The experimental verbs related to actions were selected from a corpus of 107 words. In a pilot study, these words were submitted to a group of 13 subjects. They were asked to rate the words according to their level of motor imageability.

The action verbs with the higher level of motor imageability were selected for the experiments. The verbs were matched based on word frequency and length in according to Bortolini, Tagliavini and Zampolli [24]. Each verb was presented in two different verbal forms of Italian language: at the second singular personal of the future tense (i.e. “scriverai”, *you will write”*); at the second singular personal of the past tense (i.e. “scrivevi”, *you wrote*).

#### Procedure

The experiment consisted of 64 trials. The participants were positioned 60 cm opposite the computer screen and were required to recognize the verbal tense of the stimulus.

A fixation point (black cross, 1° of visual angle) appeared in the centre of the screen for 100 msec. One-hundred msec following the presentation of the fixation point, verbal stimuli were presented for 500 msec. Subjects responded by pressing either of two response buttons with their right and left hand. In half of the trials, subjects responded with the left hand to the past tense and with the right hand to the future tense and vice-versa in the other half of the trials. The inter-trial interval was of 1500 ms. Therefore, subjects completed the task in less than 3 minutes.

The order of stimuli was randomised across subjects. Reaction times (RTs) and accuracy (number of correct responses) were measured.

### Experiment 2

#### Subjects

Twenty-four healthy participants (age-range 20–35 years; education level: 12 years) took part in this study. None of them participated in experiment 1. All were right-handed, native Italian speakers.

#### Stimuli

Stimuli were divided in 4 lists. Each list included: 10 action verbs, 10 state verbs and 10 non-verbs. Also these lists of stimuli were created with the same procedure of experiment 1.

Both verbs and non-verbs were presented in the same verbal forms as in experiment 1. Reaction times (RTs) and accuracy (number of correct responses) were measured.

#### Procedure

The experimental procedure was the same as in experiment 1. The experiment was divided in two different phases: in the first phase the subjects made the task in a baseline condition; in the second phase they made the task immediately following a train of rTMS.

For each phase, subjects responded with the left hand to the past tense and with the right hand to the future tense and vice-versa in two randomised conditions. For each condition different lists of stimuli were used.

Subjects were divided in two groups: in twelve subjects, rTMS was applied over the left cerebellum, in the other twelve subjects rTMS was applied over the right cerebellum.

The order of the phases and of lists within conditions were randomised.

Each experiment was preceded by a training phase with a list of 16 verbs different from those included in the main experiment. Each item was presented in a random order with the restriction that the same item could never be presented in consecutive trials.

#### rTMS Protocol

rTMS over the lateral (left or right) cerebellum was applied using the same scalp coordinates as Theoret et al. [25] and Torriero et al. [26]–[27] (1 cm under and 3 cm left/right to the inion).

**Figure 1:**
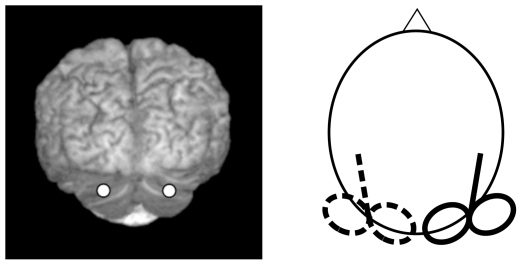
Stimulated cerebellar sites localised using Softaxic neuronavigator.

To verify the localization of the stimulated site the Softaxis navigator System was used in each subject. Although individual magnetic resonance images were not available, the Tailarach coordinates of the stimulated cortical site were automatically estimated from an MRI constructed stereotaxic template and corresponded to left and right cerebellum (Figure 1). TMS was delivered by means of a MagStim rapid magnetic stimulator, using a figure-of-eight coil (70 mm in diameter). The coil was positioned tangentially to the scalp, with the handle pointing superiorly. rTMS was applied at 1 Hz frequency for 10 min (corresponding to 600 stimuli), at an intensity of 90% of the motor threshold. Motor threshold was defined as the lowest TMS intensity (as assessed with single-pulse TMS of the contralateral motor cortex) able to induce a visible muscle twitch of the contralateral hand (i.e., ipsilateral to cerebellar stimulation) in at least 50% of a sequence of 10 consecutive trials. The task was performed immediately after the cessation of the rTMS train.

## Results

### Experiment 1

Reaction times (RTs) and accuracy were analysed with repeated measures ANOVA, with Semantic class (state verbs, action verbs, non-verbs), Tens of the verb (past, future) and Space (left, right) as factors.

#### RTs

**Figure 2:**
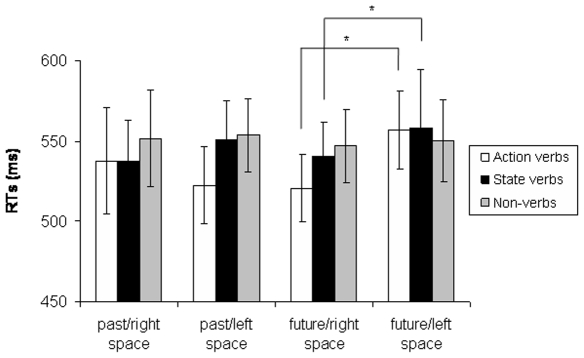
Average RTs to past tense and future tense of action verbs, state verbs and non-verbs in the left vs. right space. Asterisks indicate statistically significant differences.

We found a significant Semantic effect [F(2,46) = 3.4; p = 0.04]. RTs to action verbs were faster than RTs to state verbs (p = 0.04) and to non-verbs (p = 0.01). There was also a significant Semantic × Tense × Space interaction [F(2,46) = 6.8; p = 0.002]. When considering action verbs, RTs to future tense were faster in the right than in the left space (p<0.0001); RTs to past tense tended to be faster in the left than in the right space (p = 0.09). When considering state verbs, RTs to future tense were faster in the right than in the left space (p = 0.005); RTs to past tense were not significantly different in the left vs. right space. No significant differences in RTs to future and past tense in the right vs. left space were found for non-verbs (Figure 2).

#### Accuracy

**Figure 3:**
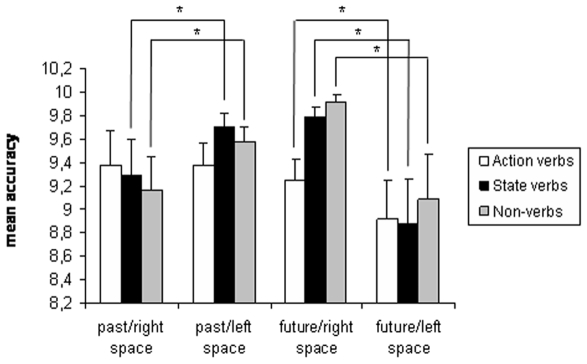
Average accuracy of responses to past tense and future tense of action verbs, state verbs and non-verbs in the left vs. right space. Asterisks indicate statistically significant differences.

We found a significant Semantic effect [F(2,46) = 5.3; p = 0.008]. Subjects were less accurate when responding to action verbs as compared with both state verbs (p = 0.01) and non-verbs (p = 0.004). There was also a significant Semantic × Tense × Space interaction [F(2,46) = 5.3; p = 0.008]. When considering action verbs, subjects were more accurate in responding to future tense in the right than in the left space (p = 0.05), while no differences were found for responses to past tense. When considering both state verbs and non-verbs, subjects were more accurate in responding to past tense in the left than in the right space (p = 0.02) and to future tense in the right than in the left space (p<0.001) (Figure 3).

### Experiment 2

Separate ANOVAs with the factor Hemisphere (left vs. right) as a between-group factor, and the factors Tense (past vs. future) and Space (left vs. right) as within-subjects factors were run on the TMS – baseline differences for each semantic class.

#### Action Verbs

**Figure 4:**
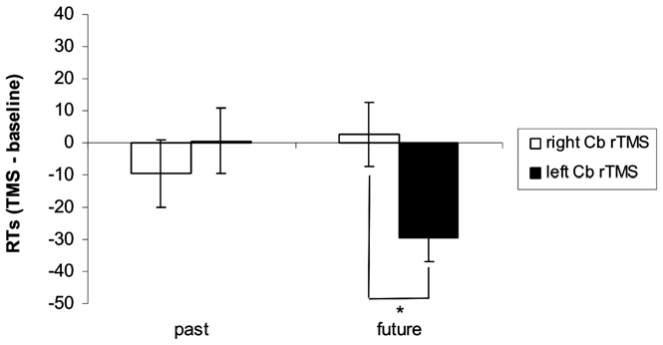
RTs to past tense and future tense of action verbs following rTMS of the right and left lateral cerebellum. Values are expressed as TMS – baseline difference. Asterisks indicate statistically significant differences.

As to RTs, a significant interaction of Hemisphere × Tense [F(1,22) = 5.1; p = 0.03] was found. rTMS of the right cerebellum interfered with processing of future tense significantly more than rTMS of the left cerebellum (p = 0.02). No significant hemispheric differences were found for processing of past tense (Figure 4).

**Figure 5:**
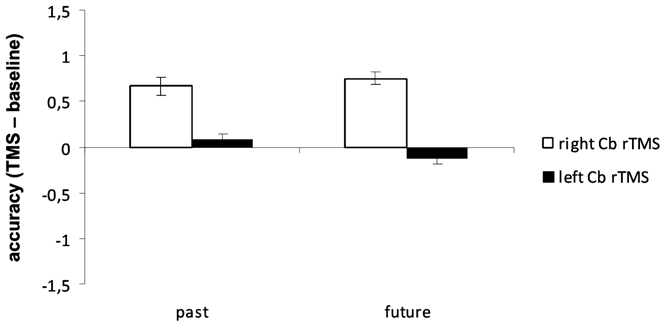
Accuracy of responses to past tense and future tense of action verbs following rTMS of the right and left lateral cerebellum. Values are expressed as TMS – baseline difference.

As to accuracy, the Hemisphere main effect tended towards significance [F(1,22) = 3.6; p = 0.07]. Right cerebellar rTMS tended to increase accuracy as compared with left cerebellar rTMS. No other significant main effects nor interactions were found (Figure 5).

#### Non-Verbs

As to RTs, ANOVA revealed a significant main effect of Hemisphere [F(1,22) = 5.6; p = 0.02]. Right cerebellar rTMS slightly interfered with subjects' performance, while the effect of left cerebellar rTMS was facilitatory. The interaction of Hemisphere × Tense only approached significance [F(1,22) = 3.1; p = 0.08].

**Figure 6:**
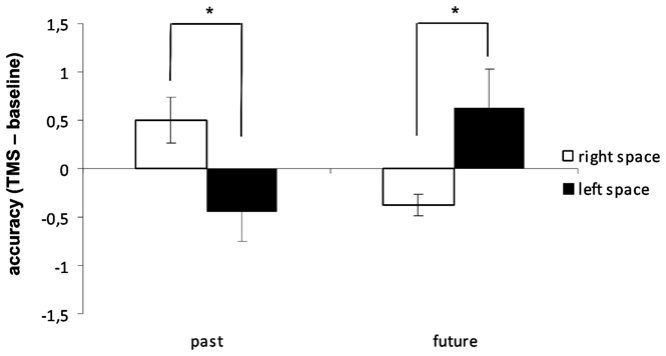
Accuracy of responses to past tense and future tense of non-verbs as a function of space (left vs. right) following rTMS of the right and left lateral cerebellum. Values are expressed as TMS – baseline difference. Asterisks indicate statistically significant differences.

As to accuracy, there was a significant interaction of Tense × Space [F(1,22) = 10.8; p = 0.003]. Regardless of the cerebellar hemisphere stimulated, rTMS decreased accuracy to past tense in the left as compared with right space (p = 0.03), and to future tense in the right as compared with left space (p = 0.02) (Figure 6).

#### State Verbs

As to RTs, no significant main effects or interactions were found.

**Figure 7:**
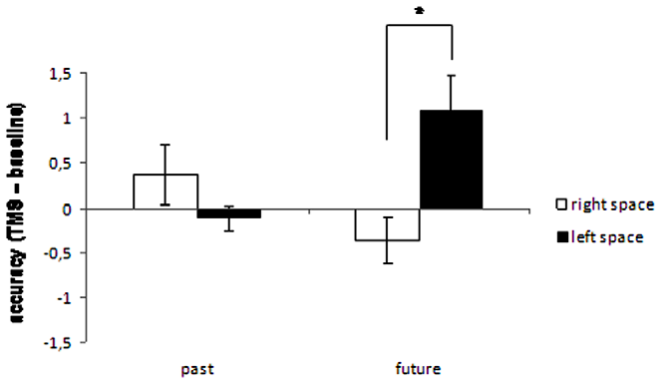
Accuracy of responses to past tense and future tense of state verbs as a function of space (left vs. right) following rTMS of the right and left lateral cerebellum. Values are expressed as TMS – baseline difference. Asterisks indicate statistically significant differences.

As to accuracy, there was a significant interaction of Tense × Space [F(1,22) = 6.4; p = 0.01]. Regardless of the cerebellar hemisphere stimulated, rTMS decreased accuracy to future tense in the right as compared with left space (p = 0.01) (Figure 7).

## Discussion

The main results of the present study show a spatial-temporal association of response codes in a task requiring to match the tense of verbs with side of space: healthy subjects are faster and more accurate in responding to future tense of a verb in the right space and to past tense of a verb in the left space.

This mapping of time with spatial factors differs according to the semantic class of the verbs: when processing action verbs, subjects are both faster and more accurate in responding to the future tense in the right space, while they are faster but not more accurate in responding to past tense in the left space. When processing state verbs, subjects are faster and more accurate in responding to future tense in the right space and more accurate in responding to past tense in the left space; when processing non-verbs, subjects are more accurate in responding to non-verbs conjugated to the future tense in the right and to the past tense in the left space, while they do not present any spatial-temporal compatibility in their response in terms of RTs. Moreover, regardless of the tense of the verb, subjects are faster but less accurate when processing action verbs as compared with state verbs and non-verbs, as if the motor imagery processes activated by action verbs have increased motor response at the expense of accuracy.

Disruption of the activity of the lateral cerebellum interferes with these spatial-temporal associations in the linguistic task. Specifically, rTMS of the right cerebellum selectively disrupts RTs to the future tense of action verbs. On the other hand, rTMS of right and left cerebellar hemispheres decreases accuracy of responses to future tense in the right space for both state verbs and non-verbs and to past tense in the left space for non-verbs.

These findings suggest that the mental time line metaphor according to which the time flows in a linear way from left to right [4], [28] could be extended to wide abstract concepts such as past and future in the linguistic domain. Processing the tense of verbs seems to be a sufficient stimulus to activate these representations, that are subsequently mapped onto spatial representations.

These results complement and extend those of Santiago and Lupianez [9], by showing that there is a spatio-temporal association (i.e. past in the left and future in the right space) even for verbs conjugated at the past and future tense. Our findings show that this spatio-temporal association is critically dependent on both semantic processes (i.e. the semantic class of the verb) and morphological processes (i.e. the morphological rules to make the past and future tense of a word). In fact, the future tense of action and state verbs mapped with the right space in terms of both RTs and accuracy; on the other hand, a significant mapping of past tense with the left and of future tense with the right space was still present in terms of accuracy for non-verbs, i.e. for stimuli that do not activate semantic processes.

Interestingly, disruption of the activity of the cerebellum interfered with either semantic or morphological processes according to the stimulated hemisphere. rTMS of the right cerebellum selectively slowed down processing of future tense of action verbs. This result suggests a critical role of action for future oriented representations, as if “moving” to the future actually required the representation of movement itself. This result is in accord with the results of Szpunar et al. [23], reporting the activation of right cerebellum and left lateral premotor cortex in subjects imagining future scenarios.

These findings complement the cognitive approach of Casasanto and Boroditsky [29] with the neuropsychological approach of Kemmerer [30] and with the theory of magnitude of Walsh [2] in indicating that action is the foundation of space-time interactions even in linguistic tasks. Casasanto and Boroditsky [29] reasoned that a determinant of the directionality of time could come from the asymmetry of space-time interactions in language. The Authors showed that the relationship between space and time in language also applies for representations of distance and duration, and suggest that our mental representations of things may be based on representations of physical experiences in perception and action.

Kemmerer [30] reported that the spatial and temporal meanings of English prepositions can be selectively impaired in different left-brain-damaged patients, suggesting that they are represented independently in the brain.

Walsh [2] indicates the action is likely to represent the missing link between different magnitudes such as space and time. Space and time are integrated metrics for action and are mapped in brain areas involved in sensory-motor transformations for action, such as the parietal and prefrontal cortices, supplementary motor area, basal ganglia and cerebellum [4], [31].

According to this premise of a critical role of motor representations in processing time, we hypothesize recruitment of a right cerebellar-left motor brain network when processing the future. This network is likely to operate in parallel with other brain circuits using past experiences to anticipate future events. On the other hand, the rTMS procedure adopted induces a long-term modulation of the excitability of cerebellar cortex, which in turn modifies the excitability of brain areas that are anatomically and functionally connected with the cerebellum.

Support for a cerebellar role in such behaviours also comes from neuropsychological studies of cerebellar patients involved in linguistic tasks. Fiez [32] and Silveri [33] reported linguistic deficits in patients with focal right cerebellar lesions as semantic retrieval deficit or expressive agrammatism. Interestingly, Silveri et al. [33] described the patient's agrammatism as due to timing deficits.

The association of spatial factors with the verbal tense of action verbs was stronger for the future tense and right space than for past tense and left space. This finding reproduces the results of previous studies, using different paradigms. In the study exploring the effects of optokinetic stimulation in time comparison tasks, the most significant effect was facilitation of the processing of longer time intervals following rightward optokinetic stimulation [3]. Similarly, Vallesi et al. [7] reported that the STARC effect was stronger in the right than in the left space. In the same line are the results of Szpunar et al. [23], documenting a right cerebellar/left premotor activation for future scenarios, while no specific activations were described for scenarios referring to the past. All these findings raise the speculation that the representation of past and future, as well as the representation of short and long time intervals, follow a metric dimension with different spatial compression factors. The representation of past, as well as the representation of short time intervals, could be more compressed and anchored to specific contextual factors as compared with that of the future [34]–[36]. In fact, future-oriented thinking has been related to episodic simulation, including planning, prediction, and remembering intentions. The present results suggest that cerebellar-motor brain networks could be part of what Schacter et al. [36] termed “the prospective brain,” whose primary function is to use past experiences to anticipate future events.

In addition to the role of the right cerebellar hemisphere for future processing of action verbs, a critical result of the present study was that rTMS of both right and left cerebellar hemispheres interfered with spatial-temporal mapping of state verbs and non-verbs.

This result suggests that in addition to the right cerebellar/left motor network involved in linking time and space through action, both cerebellar hemispheres could play a role in processing the verb conjugation per se. This finding would be in accord with previous evidence on patients with Huntington's disease, suggesting that grammatical rules may be processed by the striatum [37], [38], [39]. Since the cerebellum is also a main component of the procedural brain, we speculate that the impairment in the selection of the correct morphemes to make the past and future tense of a verb could be part of a more general impairment in selecting linguistic rules. Interestingly, this impairment follows the mental time line rule assigning a spatial-temporal compatibility of past tense with the left and of future tense with the right space.

Another, not mutually exclusive, explanation of the results is that the cerebellum plays a general role in forming sequential associations [40]. This domain-general process could be involved in establishing the mental time rule during development, selecting with experience the mapping of past with the left and of future with the right space.
